# A qualitative study evaluating experiences of a lifestyle intervention in men with prostate cancer undergoing androgen suppression therapy

**DOI:** 10.1186/1745-6215-13-208

**Published:** 2012-11-14

**Authors:** Liam Bourke, Ratna Sohanpal, Veronica Nanton, Helen Crank, Derek J Rosario, John M Saxton

**Affiliations:** 1 Department of Primary Care and Public Health, Blizard Institute, Barts and The London School of Medicine and Dentistry, Queen Mary University of London, London, E1 2AB, UK; 2 Division of Health Sciences, Warwick Medical School, University of Warwick, Coventry, CV4 7AL, UK; 3 Centre for Sport and Exercise Science, Department of Health and Wellbeing, Sheffield Hallam University, Sheffield, S10 2BP, UK; 4 Academic Urology Unit, Department of Oncology, Royal Hallamshire Hospital, University of Sheffield, Sheffield, S11 7FE, UK; 5 School of Allied Health Professions, University of East Anglia, Norwich, NR4 7TJ, UK

**Keywords:** Prostate cancer, Androgen suppression, Exercise, Diet, Rehabilitation, Patient evaluation

## Abstract

**Background:**

The severe iatrogenic hypogonadal state induced by medical castration used for treatment of prostate cancer is associated with adverse effects including fatigue, increased fracture risk, and a decrease in skeletal muscle function, which negatively impact quality of life. We have previously reported beneficial changes in healthy lifestyle behaviors, physical function and fatigue as a result of a novel combined exercise and dietary advice intervention (a lifestyle intervention) in men with prostate cancer on androgen suppression therapy (AST). The aim of this research was to conduct a qualitative evaluation of the lifestyle intervention in these men with advanced prostate cancer receiving androgen suppression therapy (AST).

**Methods:**

Twelve men with prostate cancer on AST took part in three focus groups in a UK higher education institution following the 12 week intervention. Sessions lasted between 45 and 60 minutes in duration. All discussions were audio-taped and transcribed. A framework analysis approach was applied to the focus group data. An initial coding framework was developed from *a priori* issues listed in the topic guide and extended and refined following initial familiarization with the focus group transcripts. Line by line indexing of the transcripts was undertaken iteratively to allow for the incorporation of new codes. Coded sections of text were grouped together (charted) into themes and subthemes prior to a further process of comparison and interpretation.

**Results:**

None of the participants involved in the trial were provided with information on how lifestyle changes might be beneficial to men with prostate cancer during the course of their standard medical treatment. We present novel findings that this intervention was considered beneficial for reducing anxiety around treatment and fear of disease progression. Men were supportive of the benefits of the intervention over conventional cancer survival discussion group arrangements as it facilitated peer support in addition to physical rehabilitation.

**Conclusions:**

The benefits of lifestyle changes in men with prostate cancer are not well appreciated by care providers despite a range of benefits becoming apparent. Strategies to implement exercise and dietary interventions in standard care should be further evaluated.

**Trial registration:**

Current Controlled Trials ISRCTN88605738

## Background

There are more than two million cancer survivors in the UK with this figure set to increase year on year [1]. Prostate cancer is the most frequently diagnosed malignancy in men, and medical castration through the use of androgen suppression therapy (AST) is the mainstay of treatment for advanced prostate cancer [2]. At present, nearly 50% of all men diagnosed with prostate cancer will undergo AST at some stage after diagnosis [3] and men can remain on such treatment for up to two decades [4]. The severe iatrogenic hypogonadal state induced by medical castration is associated with adverse effects including fatigue [5,6], increased fracture risk [7,8], and a decrease in skeletal muscle function [5] which negatively impact quality of life [9]. In the UK, The National Cancer Survivorship Initiative has highlighted physical symptoms as a consequence of treatment as an area of research with the highest priority [10]. We have previously reported beneficial changes in healthy lifestyle behaviors, physical function and fatigue as a result of a novel lifestyle intervention in men with prostate cancer on AST [11]. However, what is still unclear is how these men evaluate the experience of participating in the study and what recommendations should be made for integrating it into practice. Understanding patient perspectives of primary and adjunctive treatment are necessary to enable better cancer care [12]. Therefore, the aim of this study was to conduct a qualitative evaluation of a pragmatic supervised exercise program in combination with dietary advice in men with advanced prostate cancer on AST.

## Methods

### Patient details

Following ethical approval from South Sheffield Research Ethics Committee, sedentary men with histologically confirmed, T3-T4 prostate cancer who had been receiving AST for at least six months were identified from outpatient clinics and randomized either to a combined exercise and dietary advice intervention or to standard care. Those with unstable angina, uncontrolled hypertension, recent myocardial infarction, pacemakers, and painful or unstable bony metastases and those already undertaking regular physical activity (men engaging in purposeful exercise or physical activity of at least a moderate intensity for 30 minutes or more, three times per week) were excluded. Written informed consent was obtained from each participant. Randomization was carried out remotely, using nQuery statistical software (nQuery Advisor 6.01; Statistical Solutions).

### Trial details

Details of the intervention have been published elsewhere previously [11]. Briefly, in addition to a tapered supervised exercise program, a nutrition advice pack encouraging a reduction of saturated fat and refined carbohydrate and an increase of dietary fiber intake with moderation of alcohol was provided and small-group healthy eating seminars, lasting approximately 15 to 20 minutes, were carried out fortnightly. The men in the lifestyle intervention group showed improvements in exercise behavior (*P* <0.001), dietary fat intake (*P* = 0.001), total energy intake (*P* = 0.005), fatigue (*P* = 0.002), aerobic exercise tolerance (*P* <0.001) and muscle strength (*P* = 0.033) compared with standard care controls.

### Focus group details

Three to six months following the intervention, patients were invited to attend a focus group to share their experiences of the lifestyle intervention retrospectively. Three separate sessions involving a total of 12 men took place in a private conference room at Sheffield Hallam University. During the intervention men had been encouraged to attend the lifestyle program in groups to facilitate social support and inter-individual encouragement. It was felt that preserving the group structure would be a valid approach to evaluating the experiences of these men during the trial. One of the advantages of focus groups over individual interviews is the group dynamics, that is, the type and range of data generated through the social interaction of the group, can be deeper and richer than those obtained from one-on-one interviews [13]. In particular, we were keen to see how men would interact during the focus group. All men were reassured at the start of the session that any views expressed in the focus group would remain confidential. At the start of the session, men were provided with a briefing, reminding them *inter alia,* that there were no ‘right’ or ‘wrong’ answers to the questions and that constructive criticism was a valued part of the process. Care was taken by both facilitators to ask men to expand on areas of group discussion without using overtly leading questions. The topic guide was used to introduce areas for open discussion rather than to coerce the participants into specific answers. As this was not an externally funded study, men were not financially compensated for expenses incurred while taking part. Sessions lasted between 45 and 60 minutes in duration. The study authors (LB and HC) facilitated discussion around a topic guide (see Table 1) designed to explore both process and intervention outcomes. All discussions were audio-taped and transcribed before analysis.

**Table 1:** Topic guide for focus groups

**Process evaluation of the lifestyle intervention**	**Outcomes from the lifestyle intervention**
• Reasons for participation and reasons for apprehension	• Perceived benefits of the lifestyle intervention
• Structure of the supervised exercise sessions	• Perceived problems with the lifestyle intervention
• Engaging with the independent exercise sessions	• Communication with health professionals
• Frequency, intensity and duration of exercise sessions	• Burden of disease and treatment side-effects
• Contact with exercise specialists	
• Comparison to a commercial gym environment	
• Barriers to exercising	
• Format of the dietary intervention	
• Overall duration of the intervention	
• Support from peers/family	
• Comparison to conventional discussions groups	
• Burden of trial assessments	
• Recommendations for the design of future lifestyle interventions	

### Thematic framework analysis

The steps of the thematic framework analysis [14,15] were as follows. The authors firstly read and re-read the transcripts several times to become familiar with the data. The second stage involved noting salient phrases from the data. The thematic coding framework was then developed from a list of *a priori* issues listed in the topic guide and the areas identified in stage two. Inductive coding of the transcripts was conducted line by line.

Comparison of quotes was done both within and between the three transcripts. During this part of the analysis, a constant reference was made to the study aim. Thematic categories were further developed by comparing data within a category and by constant comparison of the data across categories. This was done to ensure the interpretations remain grounded in the data. These categories were defined, shared and discussed between the three authors (LB, RS and VN). Authors LB and RS conducted parallel coding with a senior qualitative researcher (VN) as a quality control measure. In addition, to making sense of individual quotes, this stage included looking at the relationship between the quotes and the links between the data as a whole, to provide explanations for the findings and overarching themes. Hence, after an analysis of the transcripts, data were categorized into these *a priori* themes, or new themes were constructed.

## Results

The themes that emerged can be broadly categorized into two overarching categories of a process evaluation of the trial and outcomes from taking part in the intervention. Descriptive quotes from focus groups (FG1, FG2, FG3) are also presented to illustrate the emergent themes. Each is now considered.

### Process themes

#### Motivations for taking part in the study

Participants were thankful for the invitation to take part in the research as it was a potential way to get back into regular exercise. The feeling of giving something back to the staff that helped treat participants with their condition and the feeling of contributing to improved treatment for ‘future patients’ appeared to suggest an altruistic reason for participation:

*‘…*I jumped at it. I’ve always tried to keep myself reasonably fit but I’ve just lapsed that little bit over the past few months for various reasons and it was a way to get on to the exercise part of it*…’* (FG3)

*‘*Every tiny bit of knowledge is beneficial to future patients*.’* (FG2)

#### Views about the supervised group design of the program

The university rehabilitation suite as a venue to exercise was a popular choice as it appeared to motivate the men to work hard and ‘get stuck in’. The participants unanimously wanted to exercise with each other and did not want to involve or include their wives particularly. This could suggest that the exercise facility was seen as a particularly ‘male’ environment. Given that these men were all hypogonadal, the presence of their wives could compromise a shared sense of masculinity:

‘When I used to come through those doors I used to think, now then we are going to get stuck in today! I want to try and do better than I did last time. ‘(FG1)

‘Would prefer just us.’ (FG1)

‘No, I think being away from them [men’s wives] is good, you need a rest from them every now and again. I don’t mind banter and nagging. But I think you would get through more without them.’ (FG2)

#### Perceived benefits of the social interaction within the group-based program

Participants found that being in the company of others living with a similar condition and exercising together was useful. Some participants found discussion about their condition with others a new and helpful experience, and were disappointed when the program ended. Participants in focus group three mentioned that they would have preferred an intervention of longer duration. The following extracts exemplify the (entirely un-coerced) peer support that was built over the course of the intervention:

‘If you are on your own you don’t know where you are going, but when you talk to other people, you have been through what they have, and it gets easier.’ (FG2)

‘I think the big disappointment is that the course ended…I would have liked it to go a little longer. We were able to discuss amongst ourselves how maybe we were coping with the bit of a health problem that we have… because being chauvinistic males we tend to keep it to ourselves and not wish to maybe discuss what the effects Zoladex can have and what condition we find ourselves in. But when I’m amongst people like this I feel safe and confident.’ (FG3)

#### Views on home-based section of the exercise program

Participants did not seem as keen to continue exercises at home as they felt they would get easily distracted by competing domestic priorities: others felt something was amiss when in the second six weeks of the program they were asked to exercise at home and visit the center only once a week:

‘At home you would probably do it once but nobody forces you the second time, but if you were coming down here [to the University] you would definitely do it. I still do it but some weeks, yes I have missed training out at home, but not very much. You get home and you say I have got to do this, but nobody forces you a second time, then it comes to the rest of the week and you think it just slips by.’ (FG2)

‘Felt as though something was missing in that second 6 weeks when we came just once a week.’ (FG1)

‘Doing it in a group [exercising], because at home, you can always do it tomorrow can’t you?’ (FG1)

#### Perceived benefits from the diet aspect of the program

The majority of participants found aspects of the diet education helpful. However, men also acknowledged that they were not able to stick to the diet advice consistently. Other statements were much more explicit in advocating its benefits:

‘Sometimes I used to buy a lot more pre-prepared meals from the supermarket. Not now. Not so much.’(FG1)

‘I think dietary advice was really good - a lot of it. I am not saying I stuck rigidly to it because I like a drink, but generally speaking I found that quite effective… I think that actually made me lose weight.’ (FG2)

#### Factors that could affect future program participation

Participation in the future would depend on whether the program continued to be perceived as beneficial. It was further highlighted that a group lifestyle intervention would be a preferable peer support structure to a conventional cancer survival discussion group format, which these men did not consider to have comparable benefits:

‘I would do it [participate in an exercise program] as a group [again], but there again you wouldn’t keep coming in if you were going to get nothing out of it. When we were doing the exercises we thought we were getting something out of it. Just having these talks [referring to group discussions], is not doing a lot of good to us. We still want a bit back.’ (FG1)

Men reported they would continue with the lifestyle changes if they perceived positive benefits in their health and that they would receive reassurances from an exercise specialist that there was continued improvement. This may have been important to the men because in their experience, meetings with the consultant had always been brief with little chance for discussion and feedback. Men appeared in favor of discussing these issues with support sisters as they did not feel so rushed at these appointments.

‘If I joined a gym and had a regime that we could cope with and that made us feel better, would it be possible to come back to see you or some other physiologist that could say yes you are still at a fitness level, it is benefitting you. You know what I mean.’ (FG2)

‘Well I tell you what those support sisters - I don’t see the consultant I just see them. I spend at least 1/2 an hour and they are never trying to rush me out of the place. They don’t just want to get rid of you: but the consultant, I have 30 seconds with him!’ (FG2)

#### Impact on exercise behavior after the intervention

Several participants joined a gym after the intervention but some men were wary about continuing after the program. Participants suggested that gyms outside of the center were not aware of the study, the program and the patient group and, as such, they were reluctant to use them. This suggests a lack of confidence in the existing community exercise facilities to understand and cater for the specific needs of men with prostate cancer on AST. In addition to this the potential cost of membership was also mentioned as a potential barrier:

‘I think I pay £28 per month which is a lot of money out of the pension.’(FG1)

‘Yes, yes, it is too expensive to go the gym [consensus of the group, yes] it would cost a fortune a week.’ (FG1)

‘I would think it [if local gyms were aware about the study and the program and that patients would be referred to another gym by researcher that] would be [beneficial]. Providing it was affordable to us.’ (FG2)

### Outcome themes

#### Disease recurrence

An unexpected finding was that men in all three groups mentioned that the intervention helped reduce fear of disease progression, adverse feelings associated with AST injections and fears about mortality. Fear of recurrence (that is, fear that cancer might return, progress or disseminate) [16,17] is a specific emotional difficulty facing some cancer survivors. It can be long standing and also negatively impact quality of life [18]. Indeed, fear of recurrence is prevalent in survivors of localized prostate cancer, and significantly predicts worse mental health related quality of life [19]. However, comparatively less is known about how it affects men on AST or strategies to ameliorate such problems:

‘It keeps your mind going and you are not thinking stupid things in the sense of I’m going to die and you can get on with your life and enjoy it..’ (FG3).

‘You don’t worry so much about prostate cancer… since I stopped exercising I found aches and pains which become more significant, whereas if I was exercising I probably wouldn’t have them*.’ (FG2)*

‘You want to get switched on a keep fit program - so you forget all about your injections and stuff like that, well I did anyway. It made me more happy.’ (FG1)

#### Communication with healthcare professionals

According to the participants, they were not told anything about lifestyle changes from nurses or consultants previously and were unsure as to whether such information should have been directly solicited. Participants, in general, felt that in addition to not having enough time during appointments with the consultant, times between follow-up appointments were also much longer than they would have preferred. Unanimously, participants were very much concerned about their prostate specific antigen (PSA) levels:

‘No [there was no mention of lifestyle changes]…I suppose one could have asked…once a year I see the consultant, yes I could ask him, but your mind seems to be on other things, i.e. what is your PSA level today rather than asking. (FG3)

‘I think the answer rests in whose hands you are with, in the hands of those nursing sisters they were a mine of information and support and then when there was some other complication arose and I saw the consultant. Afterwards, I am looking forward to going back to see them [the support sisters] again. But it is the nature of life isn’t it. All consultants are different.’ (FG2)

‘It depends if you are in control (controlled PSA), then six months isn't a long time. This gentleman is doubling every time now (i.e. PSA is doubling), that 6 months it is a hell of a long time. Now in that 6 months if it goes something daft can you bring me back again. It might be no, and you think, god if I had come after 1 month they might have done something for me. Everybody is different.’ (FG2)

‘I would like someone to tell me how it’s going - if it’s alright, if its growing, if it’s getting better…’ (FG1)

#### Benefits and drawbacks from taking part in the intervention

Participants in all focus groups perceived an improvement in their physical and psychological well-being, which is an anticipated finding given the positive quantitative results in our previous trial reports [11]. Participants were satisfied with the intensity and progression of exercises and readily engaged in goal setting. The following extracts demonstrate how psychological wellbeing and physical improvements went hand in hand:

‘I was satisfied actually but particularly with the rowing because I finished up - not with winning any record, but I felt good with doing 1,000 meters in 5 minutes.’ (FG2)

‘With exercise now I always go a bit further, I do push it a bit more. If you do the exercises there is a reward, I have managed to increase my arm strength I must admit but I do push myself a bit further than I used to.’ (FG2)

‘I feel wonderfully well…the 12 weeks in effect did me a power of good. In spite of thinking that for my age of 77 I was reasonably fit at the end of 12 weeks I know I was fit, fitter than previously.’ (FG 3)

However, there were men who believed that although exercise had benefited them in several respects, exercising would not help ease their urological side-effects. Such problems were much more of a concern to the individual in the extract below. It was their consideration that these problems are not something that a lifestyle intervention would help with:

‘I find it very difficult to say whether I feel my side effects are less. I must admit there are some side effects which I jolly well do not want to have and which I am absolutely certain exercise can do nothing about and I shall have to go back to the consultant and say, look please can we talk about this and can we make alterations. It is the [urological] side effects that are more significant things in my life it is not the fact that I have prostate cancer.’ (FG2)

## Discussion

None of the participants involved in the trial were provided with information on how lifestyle changes might be beneficial to men with prostate cancer during the course of their standard medical treatment. This analysis of post-intervention focus groups supports current evidence that lifestyle changes in men with advanced prostate cancer are beneficial [11,20,21]. Further, we present novel evidence that this intervention ameliorated anxiety around treatment and fear of disease progression. We also report men with prostate cancer have a preference for exercise rehabilitation over conventional discussion groups.

Our analysis should be interpreted in the context of relevant limitations. These views are from 12 men who were willing to be randomized in a clinical trial and as such might not be representative of all men with prostate cancer. Further research is needed to explore the preferences of men who potentially have more severe functional limitations: however, it should be noted that our recruited sample included men with metastatic disease. Also, we were unable to include all 21 men who finished the 12 week lifestyle intervention, due to limitations in their availability, although our sample of 12 was more than 50% of the cohort. The intervention was only short in duration (for example, 12 weeks); ideally cancer rehabilitation programs should be designed to provide sustained support for healthy lifestyles and investigate barriers and facilitators to sustained behavior change during implementation of longer-term (12 month) interventions. However, to the authors’ knowledge this is the first qualitative analysis of how men with prostate cancer evaluate the experience of lifestyle changes and as such represents a novel contribution to the knowledge base.

The potential for disease progression is a frequently reported concern for cancer survivors [22] and this can persist for more than five years after diagnosis [23]. Indeed, PSA levels were a persistent concern for these men. Fear of recurrence/progression has been theorized to be variable in its intensity and potentially exacerbated by imminent treatment follow-up appointments or medical check-ups [24]. Men in the focus groups reported that being involved in the rehabilitation program helped take the focus away from fear of cancer recurrence and also the unpleasant feelings associated with Zoladex injections. This finding could support the implementation of the lifestyle interventions outside of a hospital setting, possibly in the community or at a dedicated rehabilitation facility where contact with exercise specialists can be facilitated. Indeed, the supervision of exercise sessions enabled the negotiation of individually tailored goal setting between the exercise physiologist and the participants, with appropriate continuous re-evaluation. Such strategies used in conjunction with feedback to guide training progression are well recognized as important strategies to help program adherence and also promote fitness gains [25,26]. Evidence from the focus groups indicates that the men felt this process of setting and achieving goals meant they were able to exert or ‘push’ themselves further than expected. This qualitative data reported in the focus groups provides a useful context from understanding some of the quantitative findings of the trial [27]. Indeed, we reported significant improvements in aerobic exercise tolerance, muscular strength and functional capacity as a result of the lifestyle intervention and clinically relevant improvements in fatigue perception [11].

Advice regarding the benefits of lifestyle changes in men with prostate cancer, was not provided as part of standard Urology follow-up care. As the results of this analysis have demonstrated, supportive evidence in favor of healthy lifestyle changes (in parallel with similar findings elsewhere) [20,21], should be passed on during routine clinical practice. These men were strongly supportive of an exercise or lifestyle-based rehabilitation program rather than a discussion and support group. Although previous reports from a meta-analysis have indicated that psychosocial interventions (including guided support groups) are effective at improving overall quality of life in cancer survivors, [28] our findings are similar to previous qualitative research in other cancer cohorts indicating that rehabilitation programs are often the patient’s preference [29,30]. It is appropriate to draw parallels with the intervention format and the group cohesion often associated with self-help or support groups [31,32]. Men reported a sense of equality, confidence and peer support and this was not necessarily an anticipated component of the intervention design. As such, any discussion about disease processes or treatment adverse effects was developed without coercion, and hence at a socially acceptable and comfortable pace during the group sessions [30]. Furthermore, it should be noted that in contrast to other evidence in cancer cohorts [33] these men expressed that they preferred not to have spousal support during the rehabilitation program and that a group demographic consisting exclusively of men with prostate cancer was also a preference.

Accruing observational evidence suggests that habitual exercise could reduce disease progression and mortality in men with prostate cancer. [34,35] If this is to be tested through interventions in clinical trials, future research should consider that although men found this short-term experience beneficial, the mechanisms for provision of rehabilitation facilities need to be considered. Commercially available gym memberships were judged too expensive by these men, many of whom subsist from a pension. Long-term rehabilitation programs might have to be delivered using existing health care resources. Alternatively, community exercise facilities with subsidized membership schemes would be appealing. A behavior change support structure will also likely be required to facilitate long-term lifestyle change adherence, which could potentially be delivered at monthly intervals through existing consultations with cancer nurse specialists. Such consultations should include evaluation of progress and re-assessment of lifestyle goals as appropriate.

## Conclusions

None of the participants involved in this study were provided with information on how lifestyle changes might be beneficial to men with prostate cancer during the course of their standard medical treatment. We present novel findings that this intervention was considered beneficial for reducing anxiety around treatment and fear of disease progression. Men were supportive of the benefits of the intervention over conventional discussion group arrangements as it facilitated peer support in addition to physical rehabilitation. The benefits of lifestyle changes in men with prostate cancer are not well appreciated by care providers despite a range of benefits becoming apparent. Strategies to implement exercise and dietary interventions in standard care should be evaluated.

## Competing interests

The authors declare they have no competing interests.

## Authors’ contributions

LB, HC and JS performed data collection. LB, RS and VN performed the analysis and all authors contributed to manuscript drafting. All authors read and approved the final manuscript.

## Role of Funding

This study was funded internally by Sheffield Hallam University.
